# Editorial: Feminist methodologies in research on violence, displacement, and power

**DOI:** 10.3389/frma.2026.1774316

**Published:** 2026-04-08

**Authors:** Alina Potts, Anny Modi

**Affiliations:** 1 The Global Women's Institute, George Washington University, Washington, DC, United States; 2 Afia Mama Asbl, Kinshasa, Democratic Republic of Congo

**Keywords:** ethics, feminist research, forced displacement and migration, gender-based violence (GBV), humanitarian, knowledge production, research methodologies and methods, violence

This Research Topic is dedicated to the memory of our co-editor Santi Kusumaningrum, who passed away on 9 July 2023 in Jakarta, Indonesia. Santi was the Director and Principal Investigator of PUSKAPA (Center on Child Protection and Wellbeing) at the University of Indonesia, and a visionary scholar and activist whose work reshaped thinking in Indonesia and globally on how to care for and protect children while confronting the systems and power structures that cause them harm. She was deeply committed to mentorship and to supporting the next generation of child protection scholars, sharing her time, knowledge, and networks with remarkable generosity. Those of us who knew Santi as a colleague, mentor, and friend mourn her loss and remain guided by her legacy: in how we conduct research, support one another, and pursue justice with care.

This Research Topic brings together eight contributions that collectively argue for feminist methodologies not as a niche or optional approach, but as essential to producing credible, ethical, and politically honest knowledge on violence, displacement, and power. Across diverse contexts—Latin America, the Middle East, Southeast Asia—the articles in this Research Topic demonstrate how feminist epistemologies reshape what we ask, how we ask it, whose knowledge counts, and how research itself intervenes in unequal systems.

**Figure 1:**
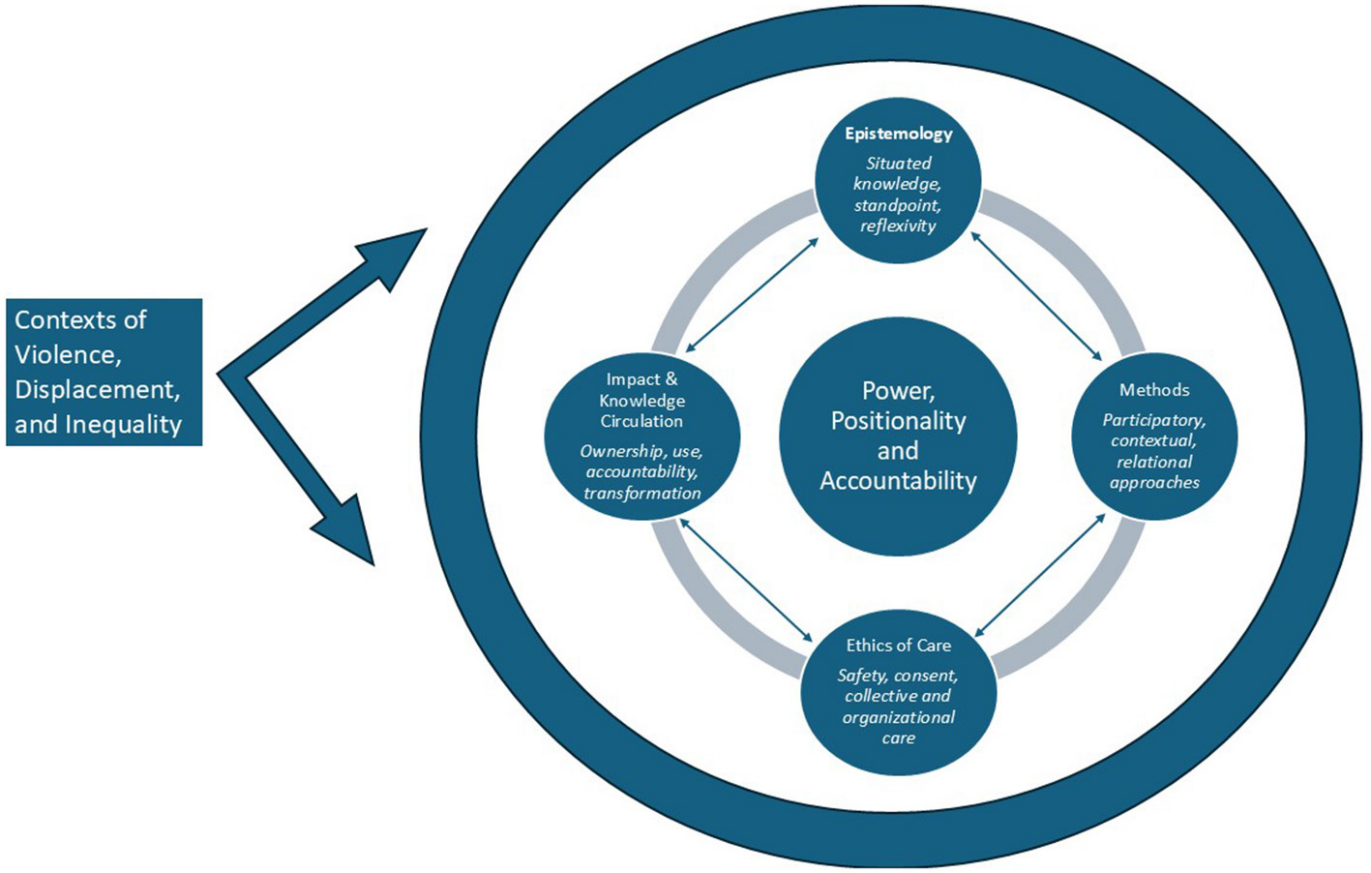
Feminist methodologies as an integrated framework for research on violence, displacement, and power: Feminist methodologies are foundational to ethical and credible research in contexts of violence and displacement. This figure highlights how feminist commitments to positionality, power analysis, and accountability shape research questions, methodological choices, researcher and participant well-being, and the circulation and use of knowledge.

Too often, studies of gender-based violence and displacement utilize approaches that prioritize extractive data collection, offer depoliticized analysis under the guise of “objectivity,” and uncover individual-level harms while obscuring the structural, historical, and relational dynamics through which violence is produced and sustained. Feminist methodologies, as illustrated across this Research Topic, offer an alternative approach grounded in reflexivity, accountability, care, and a commitment to shifting power within the research process itself (Figure 1).

Several contributions foreground feminist methods as tools for making visible forms of violence that are routinely normalized, erased, or misrepresented. Díaz-Sánchez et al. situate lethal violence against women within intersecting legal, social, and political conditions, challenging explanations that individualize risk while ignoring state accountability and structural misogyny. Similarly, Jones et al. demonstrate how participatory, feminist approaches surface women's and girls' own analyses of harm, resistance, and social constraint—insights often flattened in prevalence-driven or purely biomedical studies.

Other articles interrogate the conditions under which feminist research is conducted, particularly in fragile and conflict-affected settings. Al-Refaei offers a sobering examination of how feminist commitments to trust-building, contextual immersion, and participant-led inquiry intersect with acute security risks for researchers and participants alike. Rather than retreating from feminist practice, the article argues for more honest risk assessment, collective decision-making, and institutional responsibility, reframing “security” as relational and political rather than purely procedural.

This attention to relationality extends inward, toward those conducting research, many of whom are women. Carlson et al. challenge the normalization of burnout and vicarious trauma in violence research. Drawing on feminist ethics of care, the authors make the case that researcher wellbeing is not ancillary to methodological rigor but foundational to it—requiring structural changes in how projects are designed, resourced, and led.

Displacement and forced migration emerge as critical sites where feminist methodologies illuminate both harm and agency. Seff et al. center collective action as a mechanism through which displaced women navigate violence, rebuild social ties, and contest marginalization. By privileging women's collective experiences over individualized narratives of vulnerability, the article complicates humanitarian framings that oscillate between victimhood and resilience without attending to power.

Knowledge production itself is interrogated in the contribution by Goldmann et al., which raises pressing questions about ownership, consent, and benefit in an era of open science. Through a feminist lens, it exposes how intellectual property regimes can reproduce extractive relationships, even within ostensibly progressive research, and calls for more equitable models of knowledge circulation.

Two articles in the Research Topic engage explicitly with methodological rigor, countering persistent critiques that feminist research lacks objectivity or generalizability. Kusumaningrum et al. demonstrate how reflexivity and contextualization strengthen, rather than weaken, evidence synthesis. By making analytic decisions transparent and situating knowledge within power relations, feminist approaches enhance the credibility and relevance of reviews for policy and practice.

Finally, López Martínez et al. illustrate how feminist methodologies can be productively combined with network analysis to trace power, solidarity, and strategy within social movements. The article underscores that methodological innovation need not come at the expense of feminist commitments. Instead, it can deepen understanding of how collective action unfolds across time and space.

Taken together, the articles in this Research Topic advance three core objectives. First, they demonstrate that feminist methodologies are indispensable for accurately analyzing violence and displacement as structurally produced phenomena. Second, they show that ethics, care, and reflexivity are not add-ons but methodological imperatives that shape the quality of knowledge produced. Third, they make visible the institutional and political conditions required to support feminist research, from funding models and security protocols to authorship, data governance, and researcher wellbeing.

At a moment when research on violence is increasingly instrumentalized by being asked to produce rapid, policy-ready answers while sidestepping uncomfortable questions of power, this Research Topic insists on slowing down, listening carefully, and remaining accountable to those whose lives and struggles are the subject of study. Feminist methodologies, as these articles make clear, are not only about doing research differently; they are about reimagining what research is for, and whom it ultimately serves.

